# Clinical and economic burden of pneumococcal disease among adults in Sweden: A population-based register study

**DOI:** 10.1371/journal.pone.0287581

**Published:** 2023-07-07

**Authors:** Natalie Zarabi, Martina Aldvén, Sigrid Sjölander, Hanna Fues Wahl, Goran Bencina, Kelly D. Johnson, Sven-Arne Silfverdal

**Affiliations:** 1 MSD, Stockholm, Sweden; 2 Quantify Research, Stockholm, Sweden; 3 Center for Observational and Real-World Evidence, MSD, Madrid, Spain; 4 Center for Observational and Real-World Evidence, Merck & Co., Inc., Rahway, NJ, United States of America; 5 Clinical Sciences, Paediatrics, Umeå University, Umeå, Sweden; Faculty of Life and Allied Health Sciences, Ramaiah University of Applied Sciences, INDIA

## Abstract

Pneumococcal disease is a major cause of clinical and economic burden worldwide. This study investigated the burden of pneumococcal disease in Swedish adults. A retrospective population-based study was conducted using Swedish national registers, including all adults aged ≥18 years with a diagnosis of pneumococcal disease (defined as pneumococcal pneumonia, meningitis, or septicemia) in inpatient or outpatient specialist care between 2015–2019. Incidence and 30-day case fatality rates, healthcare resource utilization, and costs were estimated. Results were stratified by age (18–64, 65–74, and ≥75 years) and the presence of medical risk factors. A total of 10,391 infections among 9,619 adults were identified. Medical factors associated with higher risk for pneumococcal disease were present in 53% of patients. These factors were associated with increased pneumococcal disease incidence in the youngest cohort. In the cohort aged 65–74 years, having a very high risk for pneumococcal disease was not associated with an increased incidence. Pneumococcal disease incidence was estimated at 12.3 (18–64), 52.1 (64–74), and 85.3 (≥75) per 100,000 population. The 30-day case fatality rate increased with age (18–64: 2.2%, 65–74: 5.4%, ≥75: 11.7%), and was highest among septicemia patients aged ≥75 (21.4%). The 30-day average number of hospitalizations was 1.13 (18–64), 1.24 (64–74) and 1.31 (≥75). The average 30-day cost/infection was estimated at €4,467 (18–64), €5,278 (65–74), and €5,898 (≥75). The 30-day total direct cost of pneumococcal disease between 2015–2019 was €54.2 million, with 95% of costs from hospitalizations. The clinical and economic burden of pneumococcal disease in adults was found to increase with age, with nearly all costs associated with pneumococcal disease from hospitalizations. The 30-day case fatality rate was highest in the oldest age group, though not negligible in the younger age groups. The findings of this study can inform the prioritization of pneumococcal disease prevention in adult and elderly populations.

## Introduction

Pneumococcal disease (PD) is caused by *Streptococcus pneumoniae* and is a major cause of morbidity, mortality, and economic burden worldwide [1–4]. PD refers to several different types of infections and can be divided into non-invasive and invasive disease. Invasive pneumococcal disease (IPD) is defined as bacteria entering a sterile site, such as blood or cerebrospinal fluid. The burden of IPD is mainly determined by pneumococcal meningitis (PM), pneumococcal septicemia (PS) and bacteremic pneumonia. Pneumonia is the most common clinical presentation of IPD, and bacteremic pneumococcal pneumonia accounts for between 80–90% of all IPDs [5]. Non-invasive forms of PD cause pneumonia, acute otitis media, and sinusitis. In adults, non-invasive PD consists mainly of pneumococcal pneumonia (PP) [3].

The annual incidence of IPD in Sweden was quite stable between 2005 and 2016 with overall incidence of 15.5, 19.6, 11.8, and 13.4 per 100,000 in 2005, 2008, 2014, and 2016, respectively [6]. The incidence was stable again from 2015 to 2019, with an annual incidence of 13–14 cases per 100,000 inhabitants, with around 70% of reported cases occurring in adults aged 60 years and older [7]. The 30-day IPD mortality in Sweden between 2011–2013 was 10.7% among adults aged 65–74 years, but was twice as high (21.9%) among adults aged 75 years and older [8]. In 2017, 12.3% of individuals with IPD died within 30 days of infection [7]. Data on non-invasive disease is not as thoroughly collected, as it is not a notifiable disease; however, in a recent study, the incidence of PP was estimated at 20–60 cases per 100,000 person-years in 2015 for adults aged 65 years and older [4].

The burden of *S*. *pneumoniae* can to a large extent be prevented through routine vaccination. Pneumococcal conjugate vaccine (PCV) and pneumococcal polysaccharide vaccine (PPV) provide protection from IPD as well as non-invasive PP in the adult population [9]. In Sweden, the government decides on implementation of immunization programs, and in 2009 a national PCV childhood immunization program was introduced. A national immunization program covering adults with medical factors associated with a higher risk of PD (“medical risk factors” [MRFs]), as well as adults aged 65 years and older, has been discussed and is recommended by the Swedish Public Health Agency [8, 10]. In September 2021, the Swedish government announced that a national immunization program for adults aged 75 years and individuals with MRFs would be implemented starting in fall 2022 [11]. However, prior to the program start date, each region of the country was economically independent and could choose to offer vaccination free of charge to their population of choice [12].

The clinical and economic burden of PD in adults in Sweden and its relationship with MRFs is largely unknown. Even though data on the clinical burden of IPD in the adult population is available from several sources, e.g., through surveillance by the Public Health Agency (PHA), very little real-world evidence has been generated addressing this question. Furthermore, studies that examine the burden of PD mostly describe the pediatric population. A comprehensive study of the clinical and economic burden of PD in adults in Sweden could therefore be of value in the design of an efficient immunization program. The objective of this study was thus to assess the clinical and economic burden of PD in Swedish adults, describing patient characteristics, incidence rates, mortality, healthcare resource utilization (HCRU), and costs associated with PD infection. These outcomes were examined in patient groups stratified by age, the presence of MRFs, and clinical presentation.

## Methods

### Study design and data sources

This was a retrospective observational study using aggregated patient-level data from January 1, 2010 to December 31, 2019 from population-based national administrative health registers curated by the Swedish National Board of Health and Welfare (NBHW). In 2020, the outbreak of SARS-CoV-2 coronavirus resulted in a global pandemic. In Sweden, measures were implemented to reduce the spread of the infection in society and among the most vulnerable groups of individuals. This resulted in reduced incidence of other infectious diseases including PD [7]. Therefore, this study focused on the most recent pre-pandemic years (2015–2019).

Aggregated data from the Swedish National Patient Register (NPR) and the Cause of Death Register (CDR) was extracted. The NPR contains demographic, administrative, and medical information for all inpatient and outpatient specialist visits in Sweden, including information on International Classification of Disease version 10 (ICD-10) diagnosis and procedure codes. The CDR contains information on dates and causes of death. Both registers are mandatory to report to and therefore enabled nationwide coverage of the study population. Data were linked using personal identification numbers assigned to all residents and included in all administrative governmental registers in Sweden. Data linkage, aggregation, and subsequent anonymization were performed by the Swedish NBHW.

Ethical approval was obtained from the Ethical Review Authority on February 4, 2021 (reference number 2020–06487).

### Study population

This study enrolled all adult patients (≥18 years) that had at least one primary diagnosis of PD infection (for ICD-10 codes, see S1 Table) between January 1, 2015 and December 31, 2019, based on inpatient and outpatient hospital diagnoses listed in the NPR. Diagnosis of PD includes three clinical presentations: PP, PM, and PS. Whereas PM and PS are exclusively invasive, diagnosis codes of PP include both invasive and non-invasive disease, preventing separate reporting. All-cause pneumonia (ACP) codes were also included due to possible underreporting of PP and to enable comparison of incidence between PD and ACP.

The study population was stratified into three cohorts based on age. Cohorts 1 and 2 were further stratified by MRFs for PD based on the PHA’s recommendation for pneumococcal vaccination [10]. Cohort 2 was only stratified by very high risk for PD, as being 65 years and older is already considered a MRF. For specific ICD-10 and procedure codes, see S2 Table.

- **Cohort 1:** Adults aged 18–64 years
-  **Subgroup:** Any risk factor (see Table 1 for included MRFs)
- **Cohort 2:** Adults aged 65–74 years
-  **Subgroup:** Very high risk for PD (see Table 1 for included MRFs)
- **Cohort 3:** Adults aged 75 years and older

**Table 1:** Patient characteristics at first occurrence of primary diagnosis of pneumococcal disease.

Characteristic	Cohort 1: 18–64 years		Cohort 2: 65–74 years		Cohort 3: ≥75 years	
**No. of patients, n (%)**	3,285	34.2%	2,669	27.8%	3,665	38.1%
**Age group, n (%)**						
18–29	247	7.5%	-	-	-	-
30–39	467	14.2%	-	-	-	-
40–49	607	18.5%	-	-	-	-
50–59	1,026	31.2%	-	-	-	-
60–64	938	28.6%	-	-	-	-
65–74	-	-	2,669	100.0%	-	-
75–84	-	-	-	-	2,180	59.5%
≥85	-	-	-	-	1,485	40.5%
**Sex, n (%)**						
Male	1,639	49.9%	1,387	52.0%	1,819	49.6%
Female	1,646	50.1%	1,282	48.0%	1,846	50.4%
**Medical risk factors, n (%)**						
0 risk factors	1,804	**54.9%^ϕ^**	1,199	**44.9%^ϕ^**	1,543	**42.1%^ϕ^**
1 risk factor	819	**24.9%^η^**	653	24.5%	829	**22.6%^ψ^**
2 risk factors	387	**11.8%^ϕ^**	429	**16.1%^ϕ^**	723	**19.7%^ϕ^**
≥3 risk factors	275	**8.4%^ϕ^**	388	14.5%	570	15.6%
Any risk factor	1,481	**45.1%^ϕ^**	1,470	**55.1%^ϕ^**	2,122	**57.9%^ϕ^**
Very high risk of PD ^a^	724	**22.0%^ϕ^**	872	32.7%	1,177	32.1%
Immunosuppression	683	**20.8%^ϕ^**	857	32.1%	1,169	31.9%
Chronic cardiac disease	215	**6.5%^ϕ^**	484	**18.1%^ϕ^**	1,164	**31.8%^ϕ^**
Chronic respiratory disease	530	16.1%	475	**17.8%^η^**	563	**15.4%^δ^**
Diabetes mellitus	304	**9.3%^ϕ^**	441	16.5%	555	15.1%
Chronic renal failure	197	**6.0%^ϕ^**	247	**9.3%^ϕ^**	420	**11.5%^ϕ^**
Reduced lung function ^b^	234	7.1%	177	6.6%	245	6.7%
Chronic liver disease	272	**8.3%^ϕ^**	122	**4.6%^ϕ^**	74	**2.0%^ϕ^**
Organ transplant	71	2.2%	63	2.4%	18	**0.5%^ϕ^**
Functional or anatomic asplenia	51	1.6%	35	1.3%	14	**0.4%^ϕ^**
Cochlear implants	5	0.2%	4	0.2%	3	0.1%
Cerebrospinal fluid leaks	7	**0.2%^η^**	3	0.1%	1	**0.0%^ψ^**
Cystic fibrosis	2	0.1%	0	0.0%	1	0.0%

^**a**^ Includes any of the following risk factors: Functional or anatomic asplenia, Cerebrospinal fluid leak, Immunosuppression, Cochlear implants, Cystic fibrosis and Organ transplant.

^b^ Conditions that lead to reduced lung function or cough flow and stagnation of secretion.

^ϕ^ Significantly different from the two other cohorts at confidence level 95% (*P* < 0.05).

^ψ^ Significantly different from Cohort 1 at confidence level 95% (*P* < 0.05).

^δ^ Significantly different from Cohort 2 at confidence level 95% (*P*< 0.05).

^η^ Significantly different from Cohort 3 at confidence level 95% (*P* < 0.05).

### Statistical analysis and definitions

The index date was defined as the date of the occurrence of an incident pneumococcal infection during the identification period (2015–2019). A pneumococcal infection was defined as at least one recorded primary diagnosis of PD in inpatient or outpatient specialist hospital care in the identification period. An incident infection was defined as the occurrence of PD more than 30 days after a previous PD diagnosis. This meant that one patient could have several incident infections during the study period. The ICD-code for PP (J13.9) is used for both invasive and non-invasive PP, and therefore invasive disease can only be distinguished for PM and PS.

A baseline period was used to assess demographics and MRFs, and was defined as the time period from January 1, 2010, until an incident PD infection during the study period. All patients were followed for a maximum of 30 days from the index date until the end of the study period or death, whichever came first. The follow-up period was used to estimate and assess mortality and HCRU.

Descriptive statistics were computed for all PD patients, stratified by age, as numbers and percentages, at the first incident infection. The incidences of PD and ACP were calculated as the ratio between the number of incident infections and the population at risk, with 95% confidence interval (CI), per year between 2015 and 2019, and by clinical presentation. The population at risk comprised the total adult population in Sweden in each age cohort and stratification subgroup. The 30-day all-cause mortality, measured from the index date, was estimated as a case fatality rate (CFR), and reported by clinical presentation for each year between 2015 and 2019. The CFR was calculated as the percentage of incident infections resulting in death within 30 days of the index date. Subgroup analyses of separate MRFs were performed for subgroups with a sample size larger than 100 incident infections in Cohort 1: 18–64 years.

Direct costs were estimated based on cause-specific and all-cause HCRU (hospitalizations, length of stay [LOS] [13], and outpatient specialist visits) associated with incident PD infections, in the 30-day follow-up period. Costs were reported as an average cost per PD infection, overall and by clinical presentation. Unit costs for healthcare visits were sourced from the south regional price list [14] and converted to Euros (2021) [15]. Costs were estimated by multiplying each instance of resource use by the corresponding unit cost on an individual patient level.

## Results

### Patient characteristics

A total of 9,619 adults were diagnosed with PD between January 2015 and December 2019. In Table 1 the patient characteristics at the first occurrence of a primary diagnosis of PD are presented. The distribution of patients across the three cohorts was quite similar, even though Cohort 2 only accounts for an age span of 10 years. A substantial percentage of patients in Cohort 1 were designated as having very high risk of PD (22.0%), and similar percentages of Cohorts 2 and 3 (32.7% and 32.1%, respectively) had this risk level. A relatively large share of the recorded PD infections occurred in individuals without any MRFs, ranging from 42.1% in the oldest cohort to 54.9% in the youngest cohort. The most common MRF in all three cohorts was immunosuppression, observed in 20.8% to 32.1% of the patients. Cohorts 2 and 3 had very similar MRF distributions, with the next 3 most common MRFs being chronic cardiac disease, chronic respiratory disease, and diabetes mellitus. The proportion of chronic cardiac disease increased from 18.1% in Cohort 2 to 31.8% in Cohort 3, while the other 2 MRFs had similar rates of 15.1–17.8% in both cohorts. The distribution of MRFs in Cohort 1 differed, with the second most common MRF being chronic respiratory disease (16.1%), followed by 5 MRFs with similar frequencies ranging from 6.0–9.3%.

### Incidence

**Fig 1:**
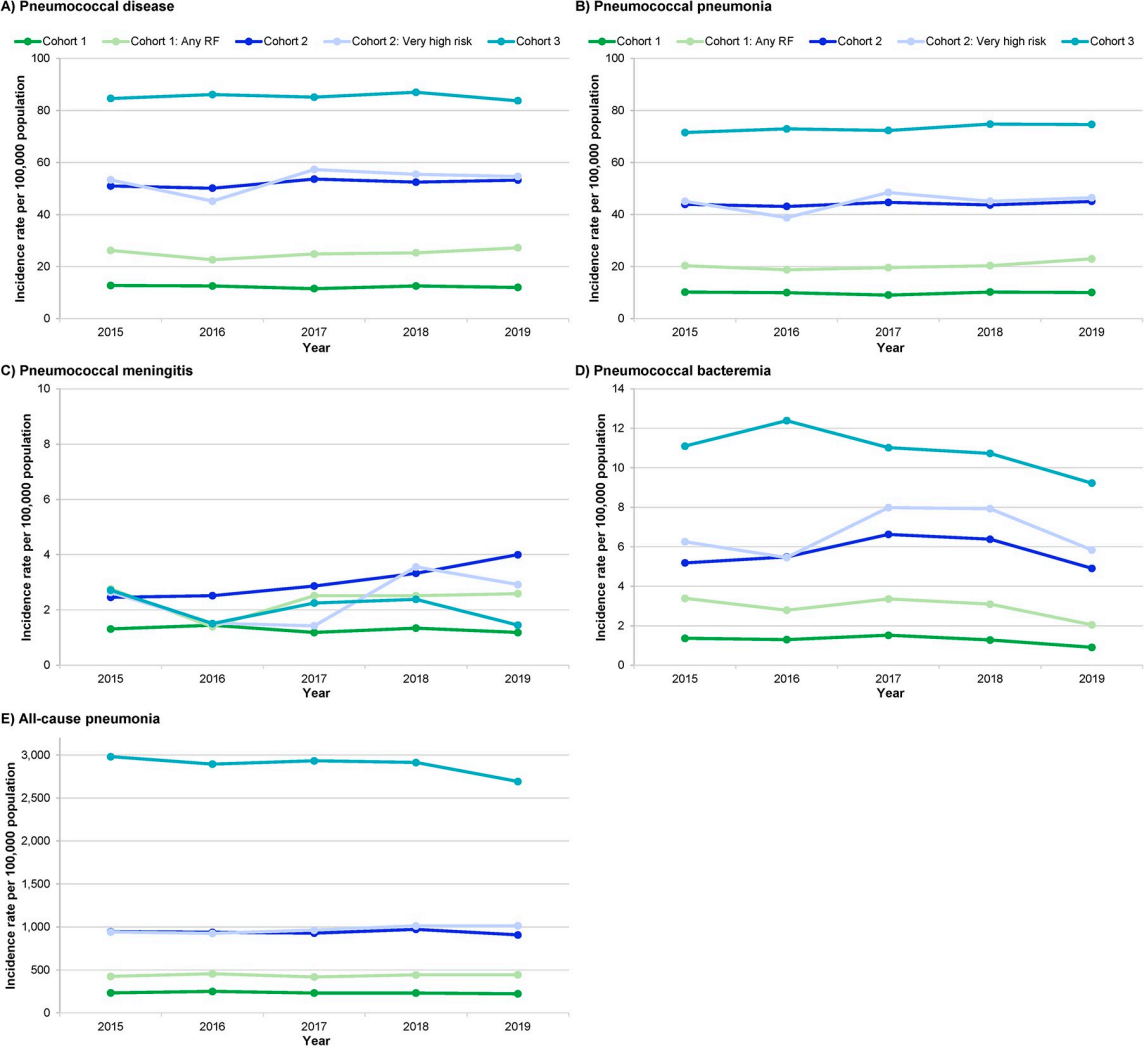
Incidence rate per 100,000 by cohort and clinical presentation.

A total of 10,391 incident PD infections were observed between January 2015 and December 2019. Of these, 3,674 occurred in adults aged 18–64 years, 2,888 in adults aged 65–75 years, and 3,829 in adults aged 75 years and older. In Fig 1 and S3 Table, the incidence rate of PD per 100,000 is presented by cohort and clinical presentation.

Several trends are noteworthy. First, and not surprisingly, the overall incidence for PD in 2015–2019 increased with age, more than doubling from Cohort 1 to Cohort 2, but not quite doubling from Cohort 2 to Cohort 3. The overall incidence for ACP (2015–2019) ranged from 234 to 2,879 per 100,000 across all the cohorts, with patterns similar to those observed for PD except that the incidence of ACP tripled between Cohort 2 and Cohort 3.

Second, of the three clinical presentations of PD, the highest incidence was observed for PP. Across age groups, PP was ≥10x more common than PM and ≥5x more common than PS.

Thirdly, we observed differential effects of MRFs in the youngest cohort vs. the older ones. In Cohort 1, having a MRF doubled the incidence of pneumonia (i.e., PD, PP, and ACP). This was also generally true for PS and PM. In contrast, in Cohort 2, the very high risk designation did not have a substantial effect on PD incidence, possibly because age is already an indicator of increased risk. Consistent with this premise, the incidence of pneumonia in Cohort 2 was approximately double that of Cohort 1 with a MRF, and the incidence of ACP more than doubled in Cohort 3 vs. Cohort 2.

Finally, PD incidence for different MRFs in the youngest cohort varied considerably, and the highest point estimates was observed in patients with chronic renal failure (124.28 per 100,000), followed by patients with chronic liver disease (81.65 per 100,00). For more details see S4 Table.

### Mortality

**Fig 2:**
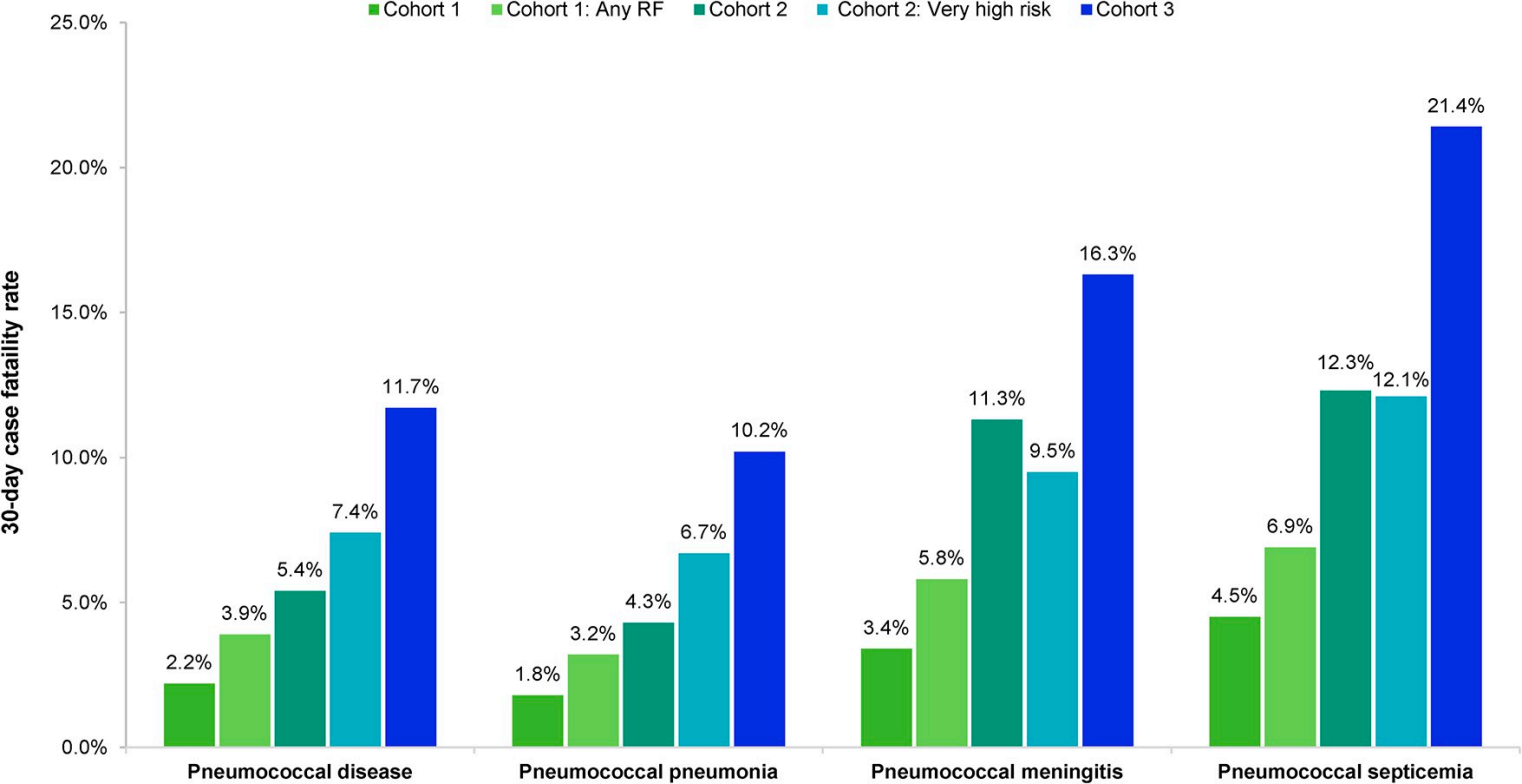
Average (all-cause) 30-day case fatality rate in 2015–2019, by clinical presentation and age cohort.

The average 30-day CFR and number of deaths in 2015–2019 by clinical presentation and age cohort are presented in Fig 2 and S5 Table. The total number of deaths from PD observed in the study period was 685, corresponding to an average of around 140 deaths per year. The CFR of PD approximately doubled from Cohort 1 to Cohort 2, and again from Cohort 2 to Cohort 3. The CFR in subgroups with MRFs was higher compared to the whole cohort for all clinical presentations of PD except for PM and PS in Cohort 2, where a slight reduction in CFR compared to the whole cohort was observed in the group with very high risk of PD. Within all age groups, the highest CFR was observed in patients with PS.

For the 30-day CFR trends by year and CFR by MRFs in Cohort 1, see S1 Fig and S4 Table.

### HCRU and costs

**Fig 3:**
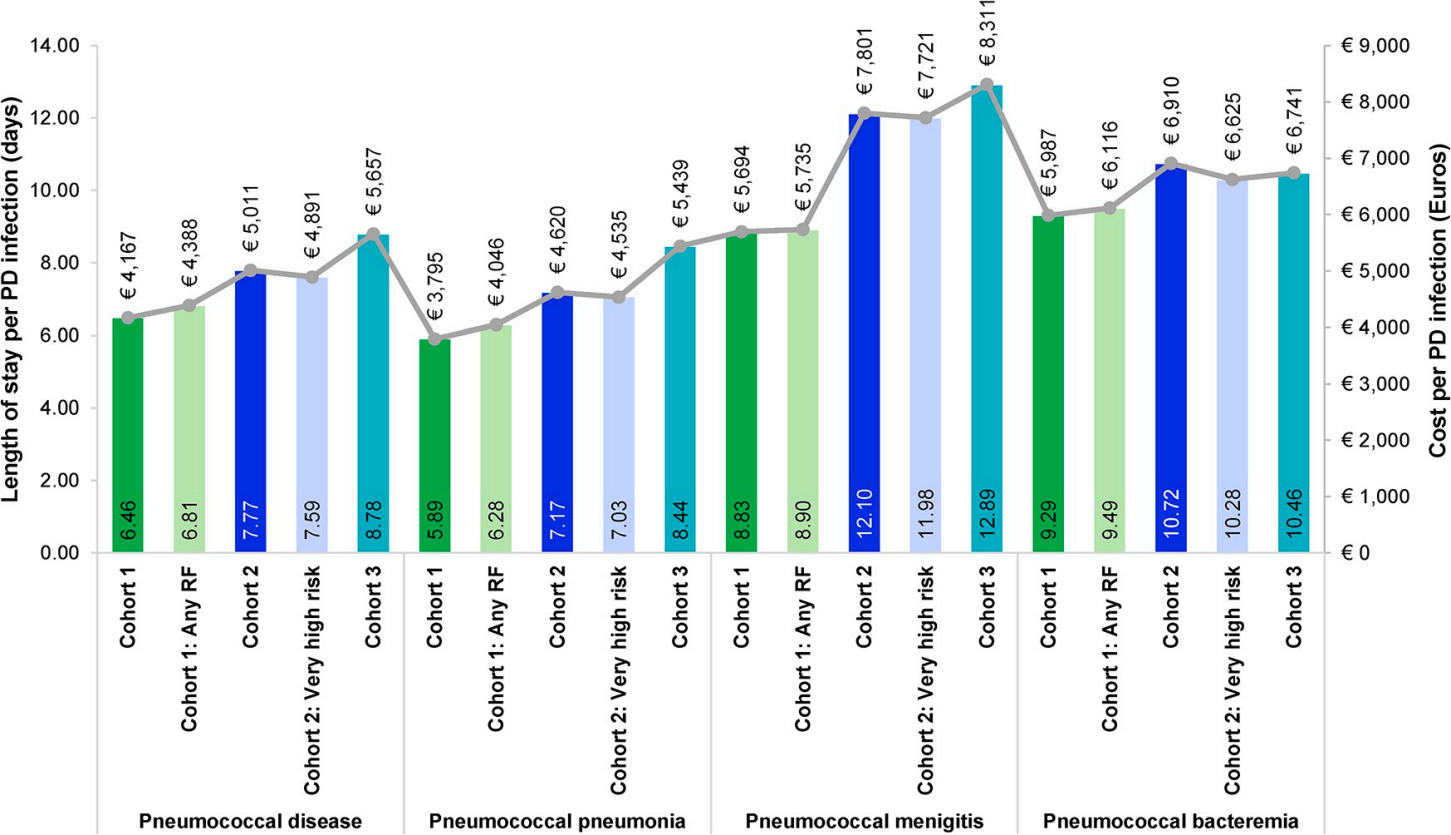
Average 30-day all-cause HCRU and costs per PD infection.

The average 30-day all-cause HCRU and cost per PD infection overall and by cohort and clinical presentation is presented in Fig 3 and S6 Table. In these age groups, 87%, 95% and 97% of incident infections respectively, required hospitalization within 30-days from index. The average number of hospitalizations and outpatient visits were quite similar across clinical presentations. Patients had on average 0.9–1.3 outpatient visits and 1.0–1.4 hospitalizations per incident infection. The LOS ranged between 5.9–8.4 days for patients with PP, 9.3–10.7 days for PS, and 8.8–12.9 days for patients with PM, with an increasing age also leading to an increased LOS, except in PS where patients aged 65–74 years had a higher point estimate than the patients aged 75 years and older. In the two older cohorts, PM caused the longest LOS, while this was caused by PS in the youngest cohort. The highest average 30-day cost per incident infection was estimated at €8,544 (inpatient and outpatient costs combined) among PM patients aged 75 years and older. Looking at PD in general, the average cost per PD infection increased with age and was highest in Cohort 3, at €5,898 per PD infection, closely followed by Cohort 2, at €5,278, and lowest in Cohort 1, at €4,467. Having a MRF was not always associated with having higher HCRU or costs.

The highest average number of hospitalizations and LOS among different MRFs in the youngest cohort was observed in patients with chronic renal failure, followed by chronic liver disease and chronic cardiac disease. For more details see S4 Table.

The total direct cost of PD, based on inpatient and specialist outpatient care between 2015 and 2019, amounted to €54.2 million, corresponding to an average yearly cost of €10.8 million, with 95% of costs being attributed to hospitalizations. Forty-two percent of the total costs could be attributed to patients aged 75 years and older, while patients aged 65–74 years accounted for 28% of the total costs.

Cause-specific HCRU was generally lower than all-cause HCRU across all clinical presentations, except for PM where the cause-specific economic burden of hospitalization was similar to the all-cause burden. Noteworthy, the share of patients requiring cause-specific hospitalization was 99% of all-cause hospitalization. For more details see S7 Table.

## Discussion

This population-based study used data from nationwide Swedish administrative health registers to estimate and describe the clinical and economic burden of PD. All adult patients with a primary diagnosis of PP, PM, or PS treated via inpatient or outpatient specialist care between 2015 and 2019 were included. Overall, both the clinical and economic burden of PD was found to increase with age and varied by the presence of MRFs.

The incidence of PD in our study was generally found to be higher than the Swedish PHA estimates for IPD, which were 40–45 per 100,000 in the age group 65 years and older (2015–2019), 14–18 per 100,000 in the age group 50–64 years, and 4 per 100,000 in the age group 20–29 years [7]. The current analysis yielded estimates of 52.5 and 87.0 per 100,000 in adults aged 65–74 and 75 years and older, respectively. This difference is most likely due to the fact that PP in this study covers both invasive and non-invasive disease. However, a significant number of the PP cases identified in this study were likely invasive, as only hospital-treated PP patients were included, whereas the majority of non-invasive pneumonia cases are treated in primary care in Sweden [8]. Thus, our incidence values likely underestimate the total number of PD cases (invasive and non-invasive combined).

A recent Swedish study estimated the incidence of ACP and PP in patients with and without comorbidities during 2005–2015 [4] and showed increased incidence with both age and the presence of comorbidities in adult patients. Our study also highlights an increase in PD incidence with age, as well as the effect of MRFs on incidence in the youngest cohort. A Norwegian study estimating hospitalized incidence in 2008–2009 [1] found that adults aged 65 years and older had the highest hospitalization rates for PS and PP. For meningitis, the incidence peaked in the age group of 60–69, which is in line with this study showing the highest PM incidence for the 65–74-year cohort.

The burden of PP is often underestimated, due to a lack of reliable diagnostic tests with the capacity to diagnose non-invasive disease [16]. *S*. *pneumoniae* is the most frequently detected pathogen in ACP, generally accepted to cause around 10–40% of adult ACP cases [17–19]. In this study, the number of incident PP cases accounted for approximately 3–5% of ACP, indicating that the burden is underestimated, though this study exclusively included cases requiring hospital and/or specialty care.

Mortality rates for invasive PP were investigated in a Swedish study in 2007–2009 with similar findings as in this study on the increasing CFR with age [20]. However, higher point estimates for mortality were found in the study by Naucler *et al*. [20], most likely due to the inclusion of invasive PP. The 30-day IPD mortality rates in Sweden between 2011–2013 were 10.7% among adults aged 65–74 years and 21.9% among adults aged 75 years and older [8]. These rates correspond well with the CFRs found for PS in this study, while lower CFRs were found for PM. The annual number of deaths (~140) found in this study is in line with a previously published study by the PHA, showing around 150 deaths per year [7].

The average 30-day LOS and associated cost of all-cause hospitalization for PD increased with age regardless of clinical presentation, with the highest LOS observed among PM patients in the two oldest cohorts. Depending on clinical presentation, the estimated LOS among patients aged 65 years and older was 40–130% higher than the average all-cause LOS in Sweden [21]. However, the median LOS was 4–6 days, which is in line with a recent report on pneumonia cases from the National Quality Register for Infectious Diseases [19]. Furthermore, a Danish study investigated the costs attributable to pneumonia among hospitalized patients and found that the costs increased with age [22], as in the current study.

More than 50% of the first recorded incident infections among patients aged 18–64 years occurred among patients without any known MRFs, and the corresponding values among the two older cohorts were over 40%. One reason for this high incidence of PD in younger adults without MRFs could be due to difficulties identifying some of the MRFs using the national health registers. Potentially missed groups include smokers, substance abusers (drugs and alcohol), and welders [10]. A Spanish study from 2012 found that one-third of adults with IPD had no MRFs but were often smokers or alcohol abusers [23]. Furthermore, in this study the identification of MRFs was based on recorded diagnoses in hospital care and thus conditions treated in primary care would be missed. However, since all MRFs can be attributed to either chronic or immunosuppressive conditions it is expected that these conditions would have been treated through outpatient specialist care at some point during the 5 years prior to PD infection. Furthermore, both the clinical and economic burden of disease increased among patients aged 18–64 years with the presence of any MRFs. On the contrary, this pattern was not noted among patients aged 65–74 years where the presence of conditions associated with very high risk of PD only marginally changed these estimates. This is in line with previous findings that the presence of MRFs has a higher impact on disease risk and burden among younger individuals than in older age groups [24]. Individuals aged 65 years and older with a MRF are also expected to be vaccinated to a higher degree, which might have influenced the outcome, however not covered by this study, as data on pneumococcal vaccination at a national level was not available at the time of study.

A recent Japanese study in PM [25], found that patients with liver and kidney diseases were significantly associated with unfavorable outcomes, including sequelae and death, compared to patients with other risk factors. This study provides an indication that both the clinical and economic burden for patients with chronic kidney failure and chronic liver disease are higher compared to patients with any MRF, however further studies are needed to determine the difference in burden.

In April 2020, Denmark was the first Nordic country to implement a pneumococcal immunization program for individuals aged 65 years and older as well as for risk groups, with the aim of reducing the number of vulnerable patients affected by serious infectious diseases other than COVID-19 [26]. In 2016, the Swedish PHA recommended vaccination, with a combination of PCV/PPV or PPV only, of individuals aged 65 years and older as well as all individuals with a MRF. Vaccination of individuals with a MRF was deemed cost-effective based on health economic analyses performed by the Swedish PHA (24). Vaccination of healthy individuals aged 65 years and older was not found to be cost-effective but is nevertheless included in the PHA recommendations [27]. In 2020, the PHA published an updated cost-effectiveness analysis for pneumococcal vaccination for the elderly in Sweden and concluded that a national immunization program starting at 65 years was unlikely to be cost-effective while a program starting at 75 years could be considered a good value [28]. In September 2021, the Swedish government announced the implementation of a pneumococcal immunization program for adults aged 75 years and for medical risk groups which started in the fall of 2022 [11]. The introduction of pneumococcal vaccination in the national immunization program will also enable future research comparing vaccinated and unvaccinated patients, as all vaccinations will be registered in the national vaccine register.

This study shows that the burden of PD among individuals aged 65–74 years with MRFs was similar to the burden in the 65–74 age group in general, with 45% of all incident infections occurring among individuals without any MRFs. This could be because older age is a stronger risk factor than MRFs, or because individuals in this age group are more often vaccinated against PD. Furthermore, it is worthwhile noting that the number of individuals with MRFs increased over the study period, from around 30% of the adult Swedish population in 2015 to 36% in 2019, in large part due to an increased number of immunocompromised patients. The average annual direct costs of PD as estimated in this study amounted to €11 million, with 58% being attributed to infections among individuals younger than 75 years, indicating that a broader pneumococcal immunization program including all individuals aged 65 years and older would prevent a large burden of disease.

In summary, the present study estimated the clinical and economic burden of PD, in the form of PP, PM, and PS diagnosed in the hospital setting, among Swedish adults stratified into three cohorts based on age and the presence of MRFs. The most common PD was pneumonia followed by septicemia and meningitis, and the overall burden of disease was found to increase with age for all outcomes investigated including incidence rates, mortality, and HCRU. The highest incidence was found in adults aged 75 years and older for all clinical presentations except meningitis. While the presence of MRFs was associated with an increased disease burden in the youngest cohort (18–64 years), this pattern was not observed in the cohort aged 65–74 years. Moreover, the proportion of patients with no MRFs recorded was unexpectedly high, constituting more than half the number of patients in the age group 18–64 years and over 40% in the two older cohorts. Overall costs were largely driven by length of hospitalization, which notably exceeded average all-cause LOS, but also depended on clinical presentation with the highest impact in meningitis. The findings of this study can support public health officials and regional stakeholders in the effective prevention of PD in the adult and elderly populations in Sweden.

## Supporting information

S1 Table Pneumococcal disease -associated diagnoses (ICD-10). (DOCX)

S2 Table Identification of underlying medical conditions / medical risk factors. (DOCX)

S3 Table Incidence rate per 100,000 by cohort and clinical presentation. (DOCX)

S4 Table Average pneumococcal disease burden 2015–2019 per risk group (>100 incident infections) in Cohort 1: Adults aged 18–64 years. (DOCX)

S5 Table Average (all-cause) 30-day case fatality rate and number of deaths in 2015–2019, by clinical presentation and age cohort. (DOCX)

S6 Table Average 30-day all-cause HCRU and costs per pneumococcal disease infection. (DOCX)

S7 Table Average total cause-specific HCRU and costs per pneumococcal disease infection. (DOCX)

S1 Fig 30-day case fatality rate for pneumococcal disease, 2015–2019 by cohort. (DOCX)
