# Supplementary material for: Clinical and economic burden of pneumococcal disease among adults in Sweden: A population-based register study
Source: PLoS One. 2023 Jul 7;18(7):e0287581. doi: 10.1371/journal.pone.0287581 (PMC10328229; doi:10.1371/journal.pone.0287581)
Supplement: S1 Table — (DOCX) [file pone.0287581.s001.docx]

**S1 Table. Pneumococcal disease-associated diagnoses (ICD-10)**

| **Clinical presentation** | **ICD-10 Codes** |
| --- | --- |
| Pneumococcal pneumonia | J13 (J13.9), J15.9 + B95.3, J18 + B95.3, J18.0 + B95.3, J18.1, J18.1 + B95.3, J18.2 + B95.3, J18.8 + B95.3, J18.9 + B95.3, J69.0 + B95.3, J85.0 + B95.3, J85.1 + B95.3, J86 + B95.3, J86.0 + B95.3, J86.9 + B95.3, J90 + B95.3, J91 + B95.3 |
| Pneumococcal meningitis | G00.1, G03.9 + B95.3 |
| Pneumococcal septicemia | A40.3, A41.9 + B95.3 |
| All-cause pneumonia | J10.0, J11.0, J11.1, J12.9, J13, J14, J15, J16.8, J17.0, J17.2, J17.3, J17.8, J18.0, J18.1, J18.8, J18.9 |
