# Supplementary material for: Clinical and economic burden of pneumococcal disease among adults in Sweden: A population-based register study
Source: PLoS One. 2023 Jul 7;18(7):e0287581. doi: 10.1371/journal.pone.0287581 (PMC10328229; doi:10.1371/journal.pone.0287581)
Supplement: S2 Table — (DOCX) [file pone.0287581.s002.docx]

**S2 Table. Identification of underlying medical conditions / medical risk factors**

| **Underlying medical condition** | **ICD-10 and procedure codes** |
| --- | --- |
| **Very high risk of pneumococcal infection:** | |
| Functional or anatomic asplenia  *- Splenectomized persons, conditions that lead to splenic dysfunction, planned splenectomy* | C26.1, C83.0D, D56, D57, D58.2, D60, D61, D73, P15.1, R16, S36.0, Q89.0, Q89.3, Q89.9 |
| Cerebrospinal fluid leaks   - *Cerebrospinal fluid leak or blood-brain barrier damage following skull surgery or trauma* | G96.0  Procedure code: AA075 |
| Immunosuppression   - *Stem cell or bone marrow transplantation. Persons with hematologic malignancies, sickle-cell disease or lung cancer.* | C00-C97, D57, Z08, Z94, B20-B23, B24.9, D00-D09, D10-D36, D37-D48, D70, D71, D80-D84, D89, Z21.9, Z51.0, Z51.1, Z51.2, Z51.5, O98.7  Procedure codes: DR010, DR008 |
| Cochlear implants | Z96.2  Procedure code: GA003 |
| Cystic fibrosis | E84 |
| Organ transplant | Z94 |
| **Increased risk of pneumococcal infection:** | |
| Chronic cardiac disease | I05-I09, I11, I13, I20-I25, I27, I34-I39, I42, I50, Q20-Q24, Q25.1 |
| Chronic respiratory disease   - *Persons with chronic obstructive*   *pulmonary disease or severe asthma* | G47.3, I27, J40-J47, J60 - J65, J68.4, J84, J96, P27 |
| Conditions that lead to reduced lung function or cough flow and stagnation of secretion   - *Persons with some chronic neurological conditions or extreme obesity* | G10, G12, G20, G24, G30, G35, G71, G72, G80, J91, E66 |
| Chronic liver disease | B15-B19, I85, K70-K77, Q44.6, R18, K65, C22, I82.0, I81.9  K50, K51 or K83.0 |
| Chronic renal failure   - *Nephrotic syndrome* | I12, I13, N00, N01, N03-N05, N17-N19, Q60, Q61, Q63, Z49, Z94.0, Z99.2 |
| Diabetes mellitus | E10-E14 |
