# Supplementary material for: Clinical and economic burden of pneumococcal disease among adults in Sweden: A population-based register study
Source: PLoS One. 2023 Jul 7;18(7):e0287581. doi: 10.1371/journal.pone.0287581 (PMC10328229; doi:10.1371/journal.pone.0287581)
Supplement: S3 Table — (DOCX) [file pone.0287581.s003.docx]

**S3 Table. Incidence rate per 100,000 by cohort and clinical presentation**

|  | **Cohort** | **No. of incident infections** | **Incidence rate per 100,000 (95% CI)** | | | | | |
| --- | --- | --- | --- | --- | --- | --- | --- | --- |
|  |  |  | **2015-2019** | **2015** | **2016** | **2017** | **2018** | **2019** |
| **PD** | **Cohort 1: 18-64 years** | 3,674 | 12.3 (11.9-12.7) | 12.7 (11.8-13.7) | 12.6 (11.7-13.5) | 11.5 (10.7-12.4) | 12.6 (11.7-13.5) | 12.0 (11.1-12.9) |
|  | *Any risk factor* | 1,648 | 25.3 (24.1-26.6) | 26.2 (23.3-29.4) | 22.6 (20.0-25.4) | 24.9 (22.2-27.7) | 25.3 (22.7-28.1) | 27.3 (24.6-30.1) |
|  | **Cohort 2: 65-74 years** | 2,888 | 52.1 (50.2-54.0) | 51.0 (46.9-55.4) | 50.1 (46.1-54.5) | 53.6 (49.4-58.1) | 52.5 (48.3-56.9) | 53.3 (49.0-57.7) |
|  | *Very high risk of PD* | 921 | 53.3 (49.9-56.9) | 53.3 (45.4-62.2) | 45.2 (38.2-53.0) | 57.3 (49.7-65.8) | 55.5 (48.1-63.7) | 54.7 (47.4-62.7) |
|  | **Cohort 3: ≥75 years** | 3,829 | 85.3 (82.6-88.1) | 84.6 (78.6-91.1) | 86.1 (80.1-92.6) | 85.1 (79.2-91.4) | 87.0 (81.1-93.3) | 83.7 (78.1-89.7) |
| **PP** | **Cohort 1: 18-64 years** | 2,957 | 9.9 (9.5-10.2) | 10.2 (9.4-11.0) | 10.0 (9.2-10.8) | 9.0 (8.3-9.8) | 10.2 (9.4-11.0) | 10.0 (9.2-10.8) |
|  | *Any risk factor* | 1,334 | 20.5 (19.4-21.6) | 20.3 (17.8-23.1) | 18.8 (16.4-21.4) | 19.6 (17.3-22.1) | 20.3 (18.0-22.9) | 23.0 (20.6-25.5) |
|  | **Cohort 2: 65-74 years** | 2,444 | 44.1 (42.4-45.9) | 43.9 (40.1-48.0) | 43.1 (39.3-47.2) | 44.7 (40.8-48.8) | 43.7 (39.9-47.7) | 45.1 (41.2-49.2) |
|  | *Very high risk of PD* | 775 | 44.9 (41.8-48.1) | 45.1 (37.9-53.3) | 38.8 (32.4-46.1) | 48.5 (41.5-56.3) | 45.1 (38.5-52.5) | 46.4 (39.8-53.8) |
|  | **Cohort 3: ≥75 years** | 3,289 | 73.3 (70.8-75.8) | 71.5 (66.0-77.5) | 72.9 (67.4-78.9) | 72.3 (66.8-78.1) | 74.8 (69.3-80.6) | 74.6 (69.3-80.3) |
| **PM** | **Cohort 1: 18-64 years** | 387 | 1.3 (1.2-1.4) | 1.3 (1.0-1.6) | 1.4 (1.2-1.8) | 1.2 (0.9-1.5) | 1.3 (1.1-1.7) | 1.2 (0.9-1.5) |
|  | *Any risk factor* | 154 | 2.4 (2.0-2.8) | 2.8 (1.9-3.9) | 1.4 (0.8-2.2) | 2.5 (1.7-3.5) | 2.5 (1.8-3.5) | 2.6 (1.8-3.6) |
|  | **Cohort 2: 65-74 years** | 168 | 3.0 (2.6-3.5) | 2.5 (1.6-3.6) | 2.5 (1.7-3.6) | 2.9 (2.0-4.0) | 3.3 (2.3-4.6) | 4.0 (2.9-5.4) |
|  | *Very high risk of PD* | 42 | 2.4 (1.8-3.3) | 2.6 (1.1-5.2) | 1.5 (0.5-3.5) | 1.4 (0.5-3.3) | 3.6 (1.9-6.1) | 2.9 (1.5-5.2) |
|  | **Cohort 3: ≥75 years** | 92 | 2.1 (1.7-2.5) | 2.7 (1.7-4.1) | 1.5 (0.8-2.6) | 2.2 (1.4-3.5) | 2.4 (1.5-3.6) | 1.5 (0.8-2.4) |
| **PS** | **Cohort 1: 18-64 years** | 380 | 1.3 (1.1-1.4) | 1.4 (1.1-1.7) | 1.3 (1.0-1.6) | 1.5 (1.2-1.9) | 1.3 (1.0-1.6) | 0.9 (0.7-1.2) |
|  | *Any risk factor* | 189 | 2.9 (2.5-3.3) | 3.4 (2.4-4.6) | 2.8 (1.9-3.9) | 3.4 (2.4-4.5) | 3.1 (2.2-4.2) | 2.0 (1.4-2.9) |
|  | **Cohort 2: 65-74 years** | 317 | 5.7 (5.1-6.4) | 5.2 (3.9-6.7) | 5.5 (4.2-7.0) | 6.6 (5.2-8.3) | 6.4 (5.0-8.0) | 4.9 (3.7-6.4) |
|  | *Very high risk of PD* | 116 | 6.7 (5.5-8.1) | 6.3 (3.8-9.8) | 5.5 (3.2-8.6) | 8.0 (5.3-11.5) | 7.9 (5.3-11.4) | 5.8 (3.7-8.8) |
|  | **Cohort 3: ≥75 years** | 487 | 10.9 (9.9-11.9) | 11.1 (9.0-13.6) | 12.4 (10.2-15.0) | 11.0 (8.9-13.4) | 10.7 (8.7-13.1) | 9.2 (7.4-11.4) |
| **ACP** | **Cohort 1: 18-64 years** | 70,100 | 234 (232-236) | 233 (229-237) | 251 (247-255) | 232 (228-236) | 232 (228-236) | 223 (219-227) |
|  | *Any risk factor* | 28,494 | 437 (432-443) | 425 (413-437) | 456 (444-468) | 419 (408-430) | 443 (432-454) | 444 (433-455) |
|  | **Cohort 2: 65-74 years** | 52,021 | 938 (930-947) | 945 (927-963) | 938 (920-956) | 929 (911-947) | 972 (954-991) | 908 (890-926) |
|  | *Very high risk of PD* | 16,818 | 974 (959-989) | 942 (908-977) | 925 (893-959) | 963 (931-996) | 1,013 (981-1,046) | 1,014 (982-1,046) |
|  | **Cohort 3: ≥75 years** | 129,182 | 2,879 (2,863-2,894) | 2,981 (2,944-3,018) | 2,894 (2,858-2,930) | 2,932 (2,897-2,968) | 2,913 (2,878-2,948) | 2,692 (2,659-2,725) |

ACP: All-cause pneumonia, PD: Pneumococcal disease, PM: Pneumococcal meningitis, PP: Pneumococcal pneumonia, PS: Pneumococcal septicemia, CI: Confidence interval
