# Supplementary material for: Clinical and economic burden of pneumococcal disease among adults in Sweden: A population-based register study
Source: PLoS One. 2023 Jul 7;18(7):e0287581. doi: 10.1371/journal.pone.0287581 (PMC10328229; doi:10.1371/journal.pone.0287581)
Supplement: S4 Table — (DOCX) [file pone.0287581.s004.docx]

**S4 Table. Average pneumococcal disease burden 2015-2019 per risk group (>100 incident infections) in Cohort 1: adults aged 18-64 years**

|  | **Number of incident infections** | **Incidence rate per 100,000 population (95%CI)** | **30-day case fatality rate, %** | **All-cause hospitalizations and costs per incident infection, mean (SD)** | | | **All-cause outpatient visits and costs per incident infection, mean (SD)** | |
| --- | --- | --- | --- | --- | --- | --- | --- | --- |
|  |  |  |  | Number of hospitalizations | Hospitalization days | Cost (€ 2021) | Number of visits | Cost (€ 2021) |
| **Cohort 1: 18-64 years** | 3,674 | 12.27 (11.88- 12.68) | 2.23% | 1.13 (0.76) | 6.46 (7.32) | 42,246 (47,861) | 1.12 (1.24) | 3,038 (3,363) |
| Any risk factor | 1,648 | 25.30 (24.10-26.55) | 3.94% | 1.15 (0.74) | 6.81 (7.60) | 44,493 (49,670) | 1.17 (1.27) | 3,170 (3,441) |
| Immunosuppression | 755 | 19.13 (17.79-20.55) | 3.58% | 1.13 (0.70) | 6.44 (7.20) | 42,110 (47,052) | 1.26 (1.26) | 3,440 (3,423) |
| Chronic cardiac disease | 241 | 42.09 (36.94-47.75) | 5.39% | 1.27 (0.85) | 8.22 (8.60) | 53,701 (56,200) | 1.07 (1.40) | 2,911 (3,797) |
| Chronic respiratory disease | 580 | 52.69 (48.49-57.16) | 4.31% | 1.14 (0.69) | 6.43 (6.92) | 42,035 (45,249) | 1.09 (1.23) | 2,973 (3,331) |
| Reduced lung function ^a^ | 272 | 28.61 (25.31-32.22) | 5.15% | 1.11 (0.74) | 6.84 (7.49) | 44,697 (48,925) | 1.08 (1.25) | 2,939 (3,394) |
| Chronic liver disease | 295 | 81.65 (72.60-91.52) | 5.76% | 1.35 (0.89) | 8.89 (8.62) | 58,140 (56,315) | 1.28 (1.32) | 3,484 (3,587) |
| Chronic renal failure | 218 | 124.28 (108.33-141.92) | 7.34% | 1.33 (0.80) | 10.33 (10.12) | 67,522 (66,157) | 1.28 (1.79) | 3,468 (4,861) |
| Diabetes mellitus | 347 | 50.78 (45.58-56.41) | 4.90% | 1.19 (0.75) | 7.42 (8.13) | 48,523 (53,144) | 1.08 (1.25) | 2,931 (3,406) |

**^a^** Conditions that lead to reduced lung function or cough flow and stagnation of secretion.
