# Supplementary material for: Clinical and economic burden of pneumococcal disease among adults in Sweden: A population-based register study
Source: PLoS One. 2023 Jul 7;18(7):e0287581. doi: 10.1371/journal.pone.0287581 (PMC10328229; doi:10.1371/journal.pone.0287581)
Supplement: S5 Table — (DOCX) [file pone.0287581.s005.docx]

**S5 Table. Average (all-cause) 30-day case fatality rate and number of deaths in 2015-2019, by clinical presentation and age cohort**

| **Cohort** | **30-day case fatality rate, % (number of deaths)** | | | |
| --- | --- | --- | --- | --- |
|  | **PD** | **PP** | **PM** | **PS** |
| **Cohort 1: 18-64 years** | 2.2% (82) | 1.8% (53) | 3.4% (13) | 4.5% (17) |
| *Any risk factor* | 3.9% (65) | 3.2% (43) | 5.8% (9) | 6.9% (13) |
| **Cohort 2: 65-74 years** | 5.4% (155) | 4.3% (104) | 11.3% (19) | 12.3% (39) |
| *Very high risk of PD* | 7.4% (68) | 6.7% (52) | 9.5% (4) | 12.1% (14) |
| **Cohort 3: ≥75 years** | 11.7% (448) | 10.2% (335) | 16.3% (15) | 21.4% (104) |

PD: Pneumococcal disease, PM: Pneumococcal meningitis, PP: Pneumococcal pneumonia, PS: Pneumococcal septicemia
