# Supplementary material for: Clinical and economic burden of pneumococcal disease among adults in Sweden: A population-based register study
Source: PLoS One. 2023 Jul 7;18(7):e0287581. doi: 10.1371/journal.pone.0287581 (PMC10328229; doi:10.1371/journal.pone.0287581)
Supplement: S6 Table — (DOCX) [file pone.0287581.s006.docx]

**S6 Table. Average 30-day all-cause HCRU and costs per pneumococcal disease infection**

| **Clinical presentation** | **Cohort** | **Hospitalizations and costs per incident infection, mean (SD)** | | | **Outpatient visits and costs per incident infection, mean (SD)** | |
| --- | --- | --- | --- | --- | --- | --- |
|  |  | Number of hospitalizations | Hospitalization days | Cost (€ 2021) | Number of outpatient visits | Cost (€ 2021) |
| **PD** | **Cohort 1: 18-64 years** | 1.13 (0.76) | 6.46 (7.32) | 4,167 (4,721) | 1.12 (1.24) | 300 (332) |
|  | *Any risk factor* | 1.15 (0.74) | 6.81 (7.60) | 4,388 (4,899) | 1.17 (1.27) | 313 (339) |
|  | **Cohort 2: 65-74 years** | 1.24 (0.75) | 7.77 (7.53) | 5,011 (4,852) | 1.00 (1.14) | 267 (307) |
|  | *Very high risk of PD* | 1.24 (0.69) | 7.59 (7.31) | 4,891 (4,711) | 1.14 (1.35) | 306 (363) |
|  | **Cohort 3: ≥75 years** | 1.31 (0.71) | 8.78 (7.24) | 5,657 (4,666) | 0.90 (1.15) | 241 (309) |
| **PP** | **Cohort 1: 18-64 years** | 1.14 (0.68) | 5.89 (6.51) | 3,795 (4,199) | 1.09 (1.20) | 291 (321) |
|  | *Any risk factor* | 1.16 (0.68) | 6.28 (6.90) | 4,046 (4,446) | 1.15 (1.22) | 308 (326) |
|  | **Cohort 2: 65-74 years** | 1.22 (0.69) | 7.17 (6.90) | 4,620 (4,447) | 0.98 (1.11) | 263 (297) |
|  | *Very high risk of PD* | 1.23 (0.64) | 7.03 (6.73) | 4,535 (4,341) | 1.13 (1.26) | 302 (339) |
|  | **Cohort 3: ≥75 years** | 1.30 (0.68) | 8.44 (6.97) | 5,439 (4,496) | 0.88 (1.04) | 236 (280) |
| **PM** | **Cohort 1: 18-64 years** | 1.01 (1.21) | 8.83 (10.22) | 5,694 (6,586) | 1.19 (1.28) | 319 (343) |
|  | *Any risk factor* | 0.95 (1.09) | 8.90 (10.45) | 5,735 (6,737) | 1.14 (1.47) | 305 (395) |
|  | **Cohort 2: 65-74 years** | 1.30 (1.14) | 12.10 (10.36) | 7,801 (6,676) | 0.98 (1.03) | 262 (275) |
|  | *Very high risk of PD* | 1.26 (1.11) | 11.98 (10.54) | 7,721 (6,794) | 1.05 (1.13) | 281 (302) |
|  | **Cohort 3: ≥75 years** | 1.37 (1.09) | 12.89 (10.38) | 8,311 (6,692) | 0.87 (0.87) | 233 (233) |
| **PS** | **Cohort 1: 18-64 years** | 1.20 (0.77) | 9.29 (9.14) | 5,987 (5,895) | 1.24 (1.46) | 332 (393) |
|  | *Any risk factor* | 1.23 (0.76) | 9.49 (9.14) | 6,116 (5,894) | 1.29 (1.40) | 345 (377) |
|  | **Cohort 2: 65-74 years** | 1.40 (0.91) | 10.72 (9.30) | 6,910 (5,998) | 1.12 (1.53) | 300 (410) |
|  | *Very high risk of PD* | 1.37 (0.89) | 10.28 (9.07) | 6,625 (5,847) | 1.34 (2.10) | 358 (563) |
|  | **Cohort 3: ≥75 years** | 1.37 (0.83) | 10.46 (8.02) | 6,741 (5,172) | 1.02 (1.73) | 273 (464) |

PD: Pneumococcal disease, PM: Pneumococcal meningitis, PP: Pneumococcal pneumonia, PS: Pneumococcal septicemia, SD: Standard deviation
