# Supplementary material for: Clinical and economic burden of pneumococcal disease among adults in Sweden: A population-based register study
Source: PLoS One. 2023 Jul 7;18(7):e0287581. doi: 10.1371/journal.pone.0287581 (PMC10328229; doi:10.1371/journal.pone.0287581)
Supplement: S7 Table — (DOCX) [file pone.0287581.s007.docx]

**S7 Table. Average total cause-specific HCRU and costs per pneumococcal disease infection**

| **Clinical presentation** | **Cohort** | **Hospitalizations and costs per incident infection, mean (SD)** | | | **Outpatient visits and costs per incident infection, mean (SD)** | |
| --- | --- | --- | --- | --- | --- | --- |
|  |  | Number of hospitalizations | Hospitalization days | Cost (€ 2021) | Number of outpatient visits | Cost (€ 2021) |
| **PD** | **Cohort 1: 18-64 years** | 0.98 (0.56) | 5.70 (6.57) | 3,673 (4,238) | 0.24 (0.55) | 64 (147) |
|  | *Any risk factor* | 0.98 (0.53) | 5.98 (6.81) | 3,852 (4,388) | 0.23 (0.55) | 60 (148) |
|  | **Cohort 2: 65-74 years** | 1.07 (0.54) | 6.77 (6.63) | 4,366 (4,273) | 0.13 (0.37) | 36 (99) |
|  | *Very high risk of PD* | 1.07 (0.50) | 6.66 (6.48) | 4,291 (4,179) | 0.11 (0.35) | 31 (94) |
|  | **Cohort 3: ≥75 years** | 1.10 (0.45) | 7.53 (6.35) | 4,852 (4,097) | 0.07 (0.27) | 19 (72) |
| **PP** | **Cohort 1: 18-64 years** | 0.99 (0.46) | 5.12 (5.63) | 3,299 (3,627) | 0.19 (0.49) | 50 (131) |
|  | *Any risk factor* | 0.99 (0.45) | 5.46 (6.03) | 3,521 (3,886) | 0.18 (0.50) | 48 (134) |
|  | **Cohort 2: 65-74 years** | 1.05 (0.47) | 6.20 (5.92) | 3,995 (3,814) | 0.12 (0.35) | 31 (94) |
|  | *Very high risk of PD* | 1.05 (0.42) | 6.12 (5.80) | 3,948 (3,736) | 0.10 (0.33) | 27 (89) |
|  | **Cohort 3: ≥75 years** | 1.09 (0.40) | 7.18 (5.98) | 4,631 (3,855) | 0.06 (0.24) | 15 (64) |
| **PM** | **Cohort 1: 18-64 years** | 0.83 (0.95) | 7.85 (9.40) | 5,061 (6,063) | 0.65 (0.80) | 174 (214) |
|  | *Any risk factor* | 0.78 (0.83) | 7.70 (9.54) | 4,965 (6,148) | 0.66 (0.80) | 176 (213) |
|  | **Cohort 2: 65-74 years** | 1.13 (0.95) | 11.38 (9.91) | 7,333 (6,390) | 0.35 (0.51) | 94 (138) |
|  | *Very high risk of PD* | 1.12 (0.99) | 11.57 (10.13) | 7,460 (6,533) | 0.43 (0.55) | 115 (147) |
|  | **Cohort 3: ≥75 years** | 1.20 (1.00) | 12.02 (10.24) | 7,750 (6,600) | 0.29 (0.48) | 79 (129) |
| **PS** | **Cohort 1: 18-64 years** | 0.92 (0.48) | 7.58 (8.31) | 4,886 (5,354) | 0.21 (0.44) | 56 (117) |
|  | *Any risk factor* | 0.93 (0.43) | 7.59 (8.13) | 4,891 (5,242) | 0.19 (0.42) | 50 (112) |
|  | **Cohort 2: 65-74 years** | 1.05 (0.51) | 7.98 (7.80) | 5,147 (5,026) | 0.14 (0.36) | 36 (97) |
|  | *Very high risk of PD* | 1.06 (0.42) | 7.83 (7.53) | 5,046 (4,855) | 0.09 (0.31) | 23 (83) |
|  | **Cohort 3: ≥75 years** | 1.05 (0.44) | 8.48 (6.92) | 5,465 (4,459) | 0.11 (0.34) | 29 (92) |

PD: Pneumococcal disease, PM: Pneumococcal meningitis, PP: Pneumococcal pneumonia, PS: Pneumococcal septicemia, SD: Standard deviation
