# Supplementary material for: Clinical and economic burden of pneumococcal disease among adults in Sweden: A population-based register study
Source: PLoS One. 2023 Jul 7;18(7):e0287581. doi: 10.1371/journal.pone.0287581 (PMC10328229; doi:10.1371/journal.pone.0287581)
Supplement: S1 Fig — (DOCX) [file pone.0287581.s008.docx]

**S1 Fig. 30-day case fatality rate for** **pneumococcal disease, 2015-2019 by cohort**
